# The RIG-I agonist M8 triggers cell death and natural killer cell activation in human papillomavirus-associated cancer and potentiates cisplatin cytotoxicity

**DOI:** 10.1007/s00262-023-03483-7

**Published:** 2023-06-25

**Authors:** Carlo Girone, Federica Calati, Irene Lo Cigno, Valentina Salvi, Valentina Tassinari, Tiziana Schioppa, Cinzia Borgogna, Ludovica Lospinoso Severini, John Hiscott, Cristina Cerboni, Alessandra Soriani, Daniela Bosisio, Marisa Gariglio

**Affiliations:** 1 Virology Unit, Department of Translational Medicine, University of Eastern Piedmont, 28100 Novara, Italy; 2 Department of Molecular and Translational Medicine, University of Brescia, Brescia, Italy; 3 Department of Molecular Medicine, Sapienza University of Rome, Rome, Italy; 4 Pasteur Institute, Fondazione Cenci-Bolognetti, Rome, Italy

**Keywords:** Human papillomavirus (HPV), RIG-I, NK cells, Cisplatin, 5’ppp-RNA

## Abstract

Although the activation of innate immunity to treat a wide variety of cancers is gaining increasing attention, it has been poorly investigated in human papillomavirus (HPV)-associated malignancies. Because these tumors harbor a severely impaired cGAS-STING axis, but they still retain a largely functional RIG-I pathway, another critical mediator of adaptive and innate immune responses, we asked whether RIG-I activation by the 5’ppp-RNA RIG-I agonist M8 would represent a therapeutically viable option to treat HPV^+^ cancers. Here, we show that M8 transfection of two cervical carcinoma-derived cell lines, CaSki and HeLa, both expressing a functional RIG-I, triggers intrinsic apoptotic cell death, which is significantly reduced in RIG-I KO cells. We also demonstrate that M8 stimulation potentiates cisplatin-mediated cell killing of HPV^+^ cells in a RIG-I dependent manner. This combination treatment is equally effective in reducing tumor growth in a syngeneic pre-clinical mouse model of HPV16-driven cancer, where enhanced expression of lymphocyte-recruiting chemokines and cytokines correlated with an increased number of activated natural killer (NK) cells in the tumor microenvironment. Consistent with a role of RIG-I signaling in immunogenic cell killing, stimulation of NK cells with conditioned medium from M8-transfected CaSki boosted NK cell proliferation, activation, and migration in a RIG-I-dependent tumor cell-intrinsic manner. Given the highly conserved molecular mechanisms of carcinogenesis and genomic features of HPV-driven cancers and the remarkably improved prognosis for HPV^+^ oropharyngeal cancer, targeting RIG-I may represent an effective immunotherapeutic strategy in this setting, favoring the development of de-escalating strategies.

## Introduction

Cancer cells and virus-infected cells share the need to escape from innate immunity to establish persistence in the host. Immune evasion is often achieved by tampering with the mechanisms regulating pathogen vs non-pathogen recognition. One of such mechanisms relies on the ability of pattern recognition receptors (PRRs) to recognize pathogen-associated molecular patterns (PAMPs), highly conserved and specific molecular structures harbored by all microorganisms, thus triggering endogenous immune responses or apoptosis. In addition to external stimuli, PRRs can also sense self-derived danger signals called damage-associated molecular patterns (DAMPs), thereby stimulating innate immunity and inflammation [1, 2].

Retinoic acid-inducible gene I (RIG-I) is an important viral sensor that induces an antiviral host response. Upon binding to 5’-tri- or diphosphate (5′ppp- or 5’pp)-single-stranded-RNAs, RIG-I triggers an antiviral signaling cascade through adaptor mitochondrial antiviral signaling (MAVS), leading to the induction of type I interferons (IFNs) and proinflammatory cytokines [3–5]. Interestingly, RIG-I signaling has been recently shown to play a key role in not only restricting viral infection but also suppressing cancer development. Specifically, RIG-I stimulation induced cell death of numerous cancer cell lines, raising the intriguing possibility that RIG-I agonists may be used as anticancer agents. In particular, the activation of RIG-I signaling in cancer cells by specific agonists, such as 5’ppp-RNAs, triggered cancer cell death programs in either a type I IFN-dependent or -independent manner. Lastly, RIG-I signaling in cancer cells mediated the activation of several innate immunity cells, including dendritic cells (DCs), natural killer (NK) cells, and CD8^+^ T cells, thereby promoting cancer cell death [6–10].

Human papillomaviruses (HPVs) are small, non-enveloped double-stranded DNA viruses responsible for the development of squamous cell carcinoma (SCC) of the anogenital and upper aerodigestive tract, with an incidence of ~ 5% among all cancers worldwide [11–13]. While virtually all cervical cancers are HPV-driven, head and neck (HN) SCCs, likely arising from HPV16-infected cells, are for the most part oropharyngeal squamous cell carcinomas (OPSCCs), with a prevalence of ~ 20%. Notably, HPV^+^ OPSCC is one of the most rapidly expanding cancers in high-income countries [14–17].

Even though cancer immunotherapies removing checkpoint restraints on adaptive immunity are gaining clinical momentum, they have so far failed to achieve widespread success in treating HPV-related cancers [14, 18]. Therefore, restoring pathways capable of triggering the innate immune response in HPV-associated tumors through production of type I IFNs and lymphocyte-recruiting chemokines is an attractive and, potentially, more effective strategy to treat this type of cancer.

Against this backdrop, PRR stimulation appears to be among the most promising therapeutic targets [6–10]. This is particularly true for HPV-infected cells where deregulated expression of the viral oncoproteins E6 and E7 leads to impaired innate immunity, thus allowing the virus to complete its life cycle and persist in the host cell, all the while boosting cell proliferation and promoting cancer development. Indeed, inhibition of E6 and E7 oncoproteins in HPV^+^ cancer cells has been shown to restore the innate immune response by interfering with the unreactive state of cancer cells that had been, until then, favoring viral persistence, cell growth, and tumorigenesis [11, 13, 19–23].

A series of reports, including some from our group, have demonstrated that the cGAS-STING signaling pathway, involved in innate immune sensing of DNA, is severely impaired in HPV^+^ cancer cells [19–23]. Specifically, we showed that HPV18 persistence in keratinocytes inhibited both type I and type III IFN production in response to DNA ligands, and that this effect was mainly due to the transcriptional repression of the cGAS-STING pathway, which was irreversible even after treatment with exogenous DNA. On the other hand, we found that the residual IFN production triggered by poly(dA:dT) was instead regulated by the RIG-I pathway, and that stimulation with the 5’ppp-RNA RIG-I agonist M8 led to transcriptional activation and secretion of type I and III IFNs in HPV^+^ cancer cells [21, 22]. As previous studies had demonstrated that RIG-I stimulation induces immunogenic cell death in human cancer cells [6–8], we asked whether RIG-I-mediated activation of innate immunity would restore the inflammatory phenotype of HPV^+^ cancer cells, making these cells more sensitive to anticancer therapy.

Here we show that treatment of HPV^+^ cancer cell lines with M8 leads to growth arrest and apoptosis, and potentiates cisplatin (Cis) cytotoxicity. The observation that intratumoral delivery of M8 leads to the recruitment of activated NK cells to the tumor microenvironment, inhibits tumor growth, and enhances the production of pro-inflammatory cytokines and chemokines, raises the possibility that this compound may be used in combination chemotherapy regimens for the treatment of human HPV-associated cancers.

## Materials and methods

### Cell culture, transfection, and treatments

HeLa cells (CRM-CCL-2™-ATCC, RRID:CVCL_0030) were grown in DMEM (Sigma-Aldrich), whereas CaSki (CRM-CRL-1550™-ATCC, RRID:CVCL_1100) and C3.43 cells (RRID:CVCL_DC63, kindly provided by Martin Kast, USA) were cultured in RPMI (Thermo Fisher Scientific), both supplemented with 10% fetal bovine serum (FBS) (Sigma-Aldrich) [24, 25]. DDX58/RIG-I knockout cells were generated with CRISPR/Cas9 technology as previously described [22]. M8 5’ppp-RNA was obtained as previously described [26] and transfected using Lipofectamine RNAiMax transfection reagent as recommended by the manufacturer (Thermo Fisher Scientific). Cells were treated with 15 µM Cis (Sandoz).

### Cell viability and apoptosis assays

Cell viability was determined by 3-(4,5-dimethylthiazol-2-yl)-2,5-diphenyltetrazolium bromide (MTT) (Sigma-Aldrich), as previously described [27].

To distinguish the type of cell death, double staining for exposed phosphatidylserine and PI exclusion was performed using the Annexin V-FITC Kit (BD Pharmingen), according to the manufacturer’s instructions. Briefly, cells were plated into 6-well plates (1.5 × 10^5^ cells/well) and transfected with M8 alone (10–500 ng/mL) or treated with a sublethal dose of Cis (15 µM). To assess the additive or synergistic effect of the two treatments, CaSki cells were transfected with M8 (10 ng/mL) and incubated in the absence or presence of 15 µM Cis for 24 h. After being harvested, cells were resuspended in annexin V/FITC and PI buffer and then analyzed by Attune™ NxT Flow Cytometer using FlowJo software (RRID:SCR_008520, Treestar). Controls of unstained cells, cells stained with Annexin V-FITC only, or cells stained with PI only were used to establish compensation and quadrants. Cells were gated according to their light-scatter properties to exclude cell debris.

#### ELISA

The cytokines secreted in the culture supernatants were analyzed using single analyte human enzyme-linked immunosorbent assay (ELISA) kits for IFN-β (DY814-05; DuoSet ELISA human IFN-β; R&D Systems), IFN-λ_1_ (DY7246; DuoSet ELISA human IL-29/IFN-λ_1_; R&D Systems), IL-6 (DY206-05; DuoSet ELISA human IL-6; R&D Systems), CCL5 (DY278; DuoSet ELISA human CCL5; R&D Systems), CXCL9 (DY392; DuoSet ELISA human CXCL9/MIG; R&D Systems), CXCL10 (DY266; DuoSet ELISA human CXCL10/IP-10; R&D Systems) and CXCL11 (DY672; DuoSet ELISA human CXCL11/I-TAC; R&D Systems) according to the manufacturer’s instructions. All absorbance readings were measured at 450 nm using a Victor X4 Multilabel plate reader (Perkin Elmer).

### Immunoblotting

Whole-cell protein extracts were prepared and subjected to immunoblot analysis as previously described [22]. Rabbit monoclonal antibody anti-PARP-1 (diluted 1:1000; 9452S, RRID:AB_2160739; Cell Signaling) was used and a mouse monoclonal antibody against α-GAPDH (diluted 1:10000; 60004-1-Ig, RRID:AB_2160739; Proteintech) was used as a control for protein loading. Immunocomplexes were detected using sheep anti-mouse (NA931; GE Healthcare) or donkey anti-rabbit (A6154; Sigma-Aldrich) immunoglobulin antibodies conjugated to horseradish peroxidase (HRP) and visualized by enhanced chemiluminescence (34580; Super Signal West Pico; Thermo Fisher Scientific) using the instrument ChemiDoc Touch Imaging System (Bio-Rad).

### In vivo tumorigenicity assay

C57BL/6 J (RRID:IMSR_JAX:000664) mice were housed under pathogen-free conditions in our animal facilities in accordance with “The Guide for the Care and Use of Laboratory Animals,” and the experimentation was approved by the Italian Ministry of Health (Agreement No. 219/2020-PR). Five-week-old mice were subcutaneously inoculated with 1.5 × 10^6^ C3.43 cells into their backs. After 12 days from the inoculum, M8 (20 µg) alone or in combination with Cis (1 mg/kg) was injected intratumorally (i.t.) and intraperitoneally (i.p.), respectively, every two days for a total of three administrations. Control mice received vehicle alone (In-vivo-JetPEI®, 101000030, PolyPlus Transfection®) or in combination with Cis. Mice were daily monitored, and tumor growth was measured over time using a Vernier caliper. The tumor was removed after 24 h from the last treatment (day 17), and its volume calculated according to the following formula: *L* × *W*2/2, where length (*L*) is the longest dimension and width (*W*) is the shortest dimension. Tumor weight was measured as well.

### Flow cytometry

Tumors were harvested, mechanically cut into small pieces, and then enzymatically treated with 1 mg/mL collagenase D (IV type, *Clostridium histolyticum*, Sigma) and 0.02 mg/mL DNase I (from bovine pancreas grade 2, Roche) at 37 °C for 30 min. The enzymatic reaction was stopped by adding EDTA (Sigma). The ensuing single-cell suspension was filtered by means of a 70-µm cell strainer and prepared for cytofluorimetric analysis. After Fc blocking, single-cell suspensions were stained with the following antibodies from Miltenyi Biotec: CD45-VioGreen (clone: REA737); NK1.1-APC (clone: PK136); CD11b-Vioblue (clone: REA592); CD3-PE (clone: REA641); CD69-PEVio770 (clone: REA937); Ly6C-PE (clone: 1G7.G10); CD4-PEVio770 (clone: REA604); CD8-Vioblue (clone: 53.6.7); and F4/80-PercPVio700 (clone: REA126). Subsequently, cell viability was determined by LIVE/Dead-633 nm (L10120) staining according to the manufacturer’s instructions (Invitrogen); negative cells were considered viable. Doublet exclusion and gating on live CD45^+^ cells were performed. The following subpopulations of CD45^+^ were identified: CD11b^+^ Ly6C^+^ (monocytes), CD11b^+^ F4/80^+^ (macrophages), CD3^+^CD4^+^ and CD3^+^C8^+^ (T cells) and NK1.1^+^CD3^−^ (NK cells). Cells were analyzed on a MACSQuant16 (Miltenyi) and analyzed with FlowJo software (RRID:SCR_008520, Treestar) [28].

### Quantitative PCR

RNA was extracted from CaSki cells or the harvested tumors using TRIzol (15596018; Thermo Fisher Scientific) according to the manufacturer’s instructions. After RNA purification, samples were treated with DNAse to remove contaminating genomic DNA. Reverse transcription was performed using random hexamers and MMLV RT (all from Thermo Fisher Scientific). The primer sequences are available upon request.

### NK cell proliferation assay

Human NK cells were purified from healthy donors’ peripheral blood by negative selection, using RosetteSep™ Human NK Cell Enrichment Cocktail (STEMCELL™ technologies). NK cell purity (CD56^+^CD3^−^) was more than 95%. Cells were labeled with the Incucyte® Nuclight Reagent (#4717 Sartorius) and grown in a 96-well plate (40,000 cells/well) in the presence of conditioned media from M8-transfected (10 ng/mL) or mock-transfected CaSki cells. Proliferation was measured by IncuCyte® Live-Cell Analysis System (Sartorius), and the data were analyzed through IncuCyte® Zoom software.

### NK cell migration assay

For migration assay, NK cells were purified as described above. Cell migration was measured using a transwell migration chamber (diameter insert: 6.5 mm, pore size: 5 μm; Costar, Cambridge, MA). As chemoattractant, conditioned medium from M8-transfected (10 ng/mL) or mock-transfected CaSki cells was added to the lower compartment. After 4 h at 37 °C, the number of migrated cells was counted using BD FACSCanto™ (RRID:SCR_018055, BD). The percentage of migrated cells was calculated as follows: number of migrated NK cells/number of input NK cells × 100.

### Cytotoxicity assay

NK cells were incubated overnight with conditioned media from M8-transfected (10 ng/mL) or mock-transfected CaSki cells, then used as effectors in a 4-h cytotoxicity assay at the E:T ratio of 20:1. CFSE-labeled CaSki cells were used as targets. After being washed once with 1% BSA in PBS, cells were resuspended in the same buffer and incubated for 20 min at 4 °C in the presence of the intercalating DNA dye 7-aminoactinomycin D (7-AAD) (5 μg/mL) (Sigma-Aldrich, St Louis, MO). Lastly, cells were fixed with 1% PFA (Sigma-Aldrich, St Louis, MO) for 20 min at 4 °C. At least 20,000 events in the CFSE^+^ gate were collected using BD FACSCanto™ (RRID:SCR_018055, BD).

### Statistical analysis

All statistical analyses were performed using Graph-Pad Prism version 8.00 for Windows. The data are represented as mean ± standard deviation (SD). For comparisons of two groups, means were compared using the two-tailed Student’s t tests. Differences were considered statistically significant at a *p*-value < 0.05.

## Results

### The RIG‐I agonist M8 strongly boosts cell death in HPV^+^ cancer cells

**Fig. 1:**
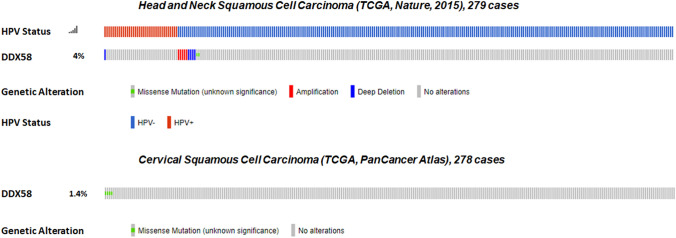
The *RIG-I/DDX58* gene is rarely mutated in HPV-associated cancers. TCGA-curated clinical data set of HNSCC (*n* = 279 samples, upper panel) and cervical squamous carcinoma (*n* = 278 samples, lower panel) were interrogated for genomic *RIG-I/DDX58* mutations. Scores for HPV status corresponding to each clinical HNSCC specimen are shown

To assess the potential applicability of RIG-I agonist-based therapy to HPV-driven cancers, we first interrogated The Cancer Genome Atlas (TCGA) dataset for *RIG-I/DDX58* gene mutations. This cohort comprises 279 HNSCCs (Fig. 1, upper panel) [29] and 278 cervical cancers (Fig. 1, lower panel) (data generated by the TCGA Research Network: https://www.cancer.gov/tcga). Among the 36 (13%) HPV^+^ HNSCC cases only one displayed a deep deletion in the *RIG-I/DDX58* gene. Among the HPV^−^ counterpart, *RIG-I/DDX58* was found amplified in only 5 cases, deep deleted in 4, and mutated (missense mutation) in 2 (Fig. 1, upper panel). As for the cervical cancers, their HPV status was unspecified. However, given that > 98% of cervical cancers are known to be HPV-driven, we decided to arbitrarily consider all these cases as HPV^+^. Among these tumors, we found that only 4 of them carried a missense mutation in *RIG-I/DDX58* (Fig. 1, lower panel). Thus, the overall mutation rate of *RIG-I/DDX58* in these two cohorts was ~ 3%, indicating that this gene is rarely lost or mutated in HPV-associated cancers.

**Fig. 2:**
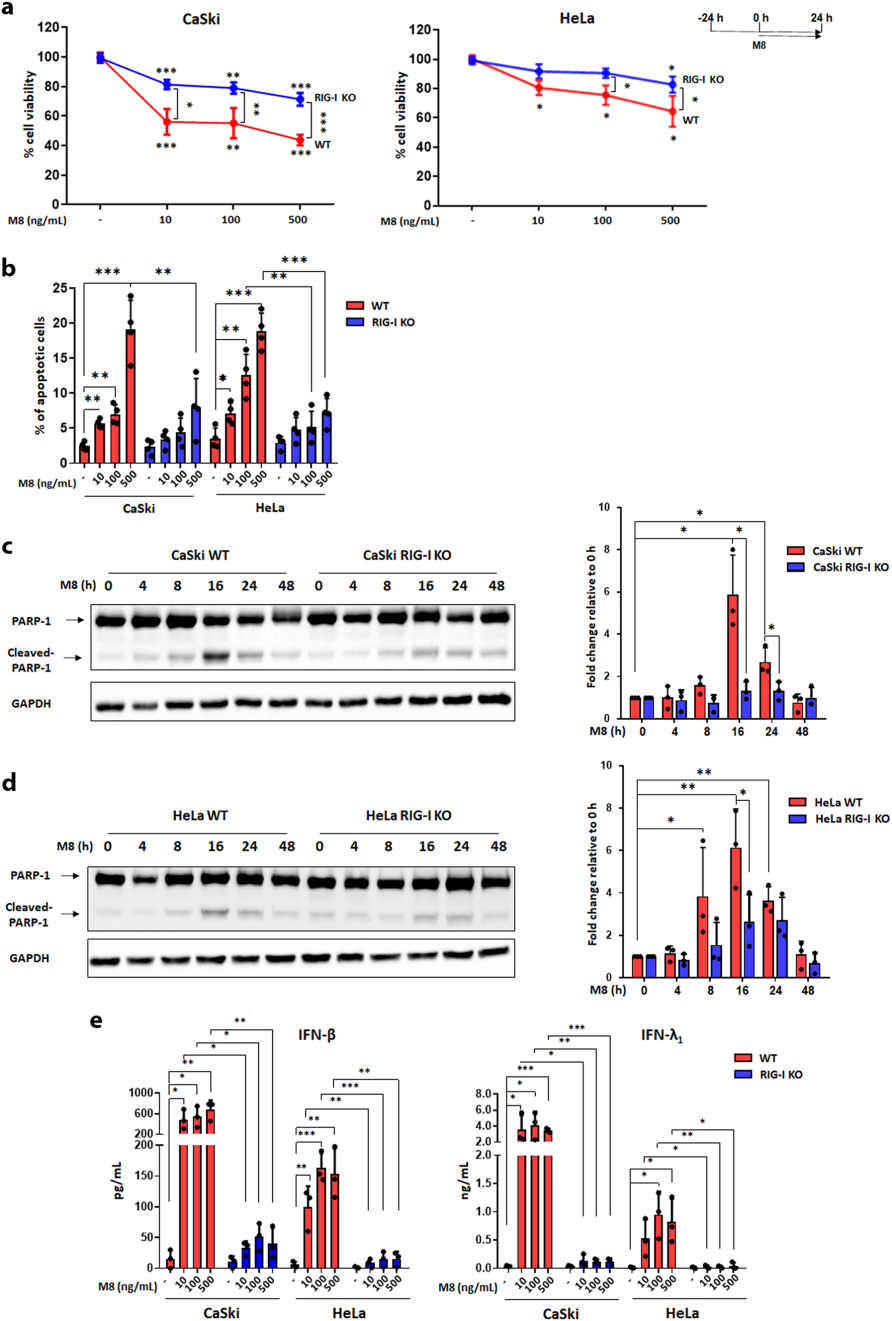
Transfection of the RIG-I agonist M8 inhibits the growth of HPV^+^ cells** a** Wild-type (WT) CaSki (left panel) or WT HeLa (right panel) or their RIG-I-deficient (RIG-I KO) counterparts were mock-transfected or transfected with increasing doses of M8 (10, 100, or 500 ng/mL) for 24 h, and then subjected to MTT assay to determine cell viability. The results are shown as mean values of biological triplicates. **b** Cells described in panel A were incubated with annexin V-FITC in a buffer containing propidium iodide (PI) and analyzed by flow cytometry. The graph shows the percentage of apoptotic cells (annexin V^+^ and PI^+^ or PI^−^ cells). Each dot represents data from a single experiment. **c, d** Total cell extracts from mock- or M8-transfected (10 ng/mL) WT or RIG-I KO CaSki (c) and HeLa (d) cells for the indicated time points were subjected to immunoblot analysis using anti-PARP-1 or GAPDH antibodies, a representative image is shown. The bands intensities for the indicated antibody were quantified by densitometry, and the ratio of the abundance of cleaved PARP-1 relative to that of GAPDH was calculated. The results are shown as mean values of biological triplicates.** e** ELISA quantitation of IFN-β and IFN-λ_1_ proteins in supernatants from the cell cultures described in panel A. Each dot represents data from a single experiment. Error bars indicate SD. *P < 0.05, **P < 0.01, unpaired t-test, ***P < 0.001, unpaired t-test

We next asked whether the activation of RIG-I RNA-sensing pathway would promote cell growth arrest and apoptosis of HPV^+^ cancer cell lines upon stimulation with the 5’ppp-RNA RIG-I agonist M8. To this end, we used CaSki and HeLa cervical carcinoma-derived cell lines harboring, respectively, an integrated HPV16 or HPV18 genome [24] and both expressing a functional RIG-I [22]. As negative control, we also used *DDX58/RIG-I* knockout (RIG-I KO) CaSki and HeLa cells, already available in our laboratory [22]. Cells were transfected in the absence or presence of increasing doses of M8 (10, 100, or 500 ng/mL) for 24 h, and then subjected to MTT assay to determine cell viability. Stimulation of WT CaSki cells with M8 at all doses (10–500 ng/mL) led to a substantial loss of cell viability (40–50%) in comparison with vehicle-transfected cells (Fig. 2a, left panel). This growth inhibition was almost halved in M8-transfected RIG-I KO vs WT CaSki cells, but still statistically significant when M8-transfected cells, both WT and KO, were compared with the corresponding vehicle-transfected cells. Similar results were obtained in M8-transfected HeLa cells (Fig. 2a, right panel), although the extent of growth inhibition was not as marked as that seen in CaSki cells. However, when we transfected RIG-I KO HeLa cells with M8, we observed a significant rescue from cell growth inhibition at the highest doses (100 and 500 ng/mL).

To determine whether M8-transfected cells were indeed undergoing apoptosis, we next performed annexin V/propidium iodide (PI) staining and flow cytometry. As depicted in Fig. 2b, the percentage of apoptotic cells in WT CaSki cells was 5.7% and 7% when M8 was used at 10 and 100 ng/mL, respectively, and it increased up to 19% when we used the highest dose (500 ng/mL). By contrast, in RIG-I KO CaSki cells, the percentage of apoptotic cells remained below 8% also at the highest dose. A similar trend was observed in WT HeLa cells, with a 19% of apoptotic cells in those cultures that were transfected with the highest dose of M8, which dropped below 7.5% in RIG-I KO HeLa cells similarly transfected. Based on these results, all the experiments hereinafter were performed by transfecting M8 at a dose of 10 ng/mL. The onset of apoptosis was further confirmed by enhanced PARP-1 cleavage observed in both M8-transfected WT CaSki at 16–24 h and WT HeLa cells at 8–16–24 h (Fig. 2c, d) [30]. Consistent with a proapoptotic activity of RIG-I, M8-transfected RIG-I KO CaSki and HeLa cells did not display any significant induction of PARP-1 cleavage.

Next, we assessed the release of both type I (IFN-β) and III IFNs (IFN-λ_1_) in the culture supernatants from WT CaSki or HeLa cells transfected or not with M8. In either cell line, the secretion of both IFN-β and IFN-λ_1_ was strongly induced upon M8 stimulation starting from the lowest dose, with negligible dose-dependent variations (Fig. 2e). Importantly, the induction of IFN levels was substantially reduced in M8-transfected RIG-I KO cell lines, suggesting that RIG-I signaling is required for transcriptional activation of IFNs.

Altogether, our findings suggest that M8-mediated activation of RIG-I signaling promotes growth arrest and apoptosis of HPV^+^ cell lines.

### Activation of RIG-I by M8 sensitizes HPV16^+^ cells to cisplatin cytotoxicity

One of the main goals of immunotherapy is that of increasing the response to standard chemotherapy to allow the development of de-escalating strategies aimed at reducing chemo-associated side effects [31–33]. Given the proapoptotic function of RIG-I signaling, we sought to determine whether M8 stimulation would sensitize HPV^+^ cancer cells to the cytotoxic effects of standard chemotherapeutic agents (e.g., Cis). As HPV16 is the most frequent HPV genotype found in HPV-associated cancers, we decided to focus hereinafter on the HPV16-based model of CaSki cells [15].

**Fig. 3:**
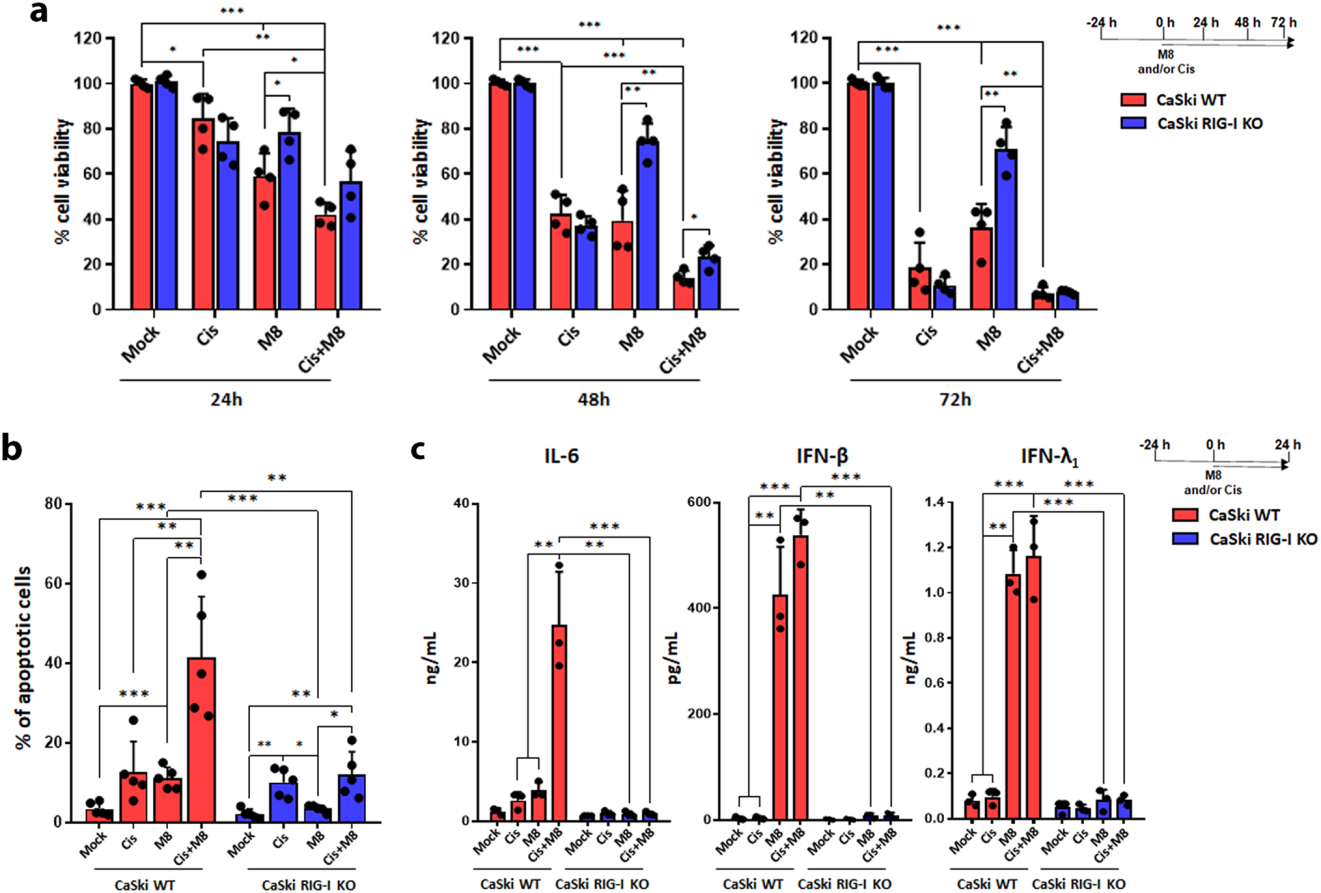
The RIG-I agonist M8 enhances the sensitivity of HPV16^+^ CaSki cells to cisplatin** a** WT or RIG-I KO CaSki cells were mock-transfected or transfected with M8 (10 ng/mL) in the presence or absence of cisplatin (Cis) (15 µM) for the indicated time points. Cell viability was determined by MTT assay. Each dot represents data from a single experiment. **b** Cells described in panel A were incubated with annexin V-FITC in a buffer containing propidium iodide (PI) and analyzed by flow cytometry. The graph shows the percentage of apoptotic cells (annexin V^+^ and PI^+^ or PI^−^ cells). Each dot represents data from a single experiment.** c** ELISA quantitation of IFN-β, IFN-λ_1_, and IL-6 proteins in the supernatants from WT or RIG-I KO CaSki cells mock-transfected or transfected with M8 (10 ng/mL) and treated or not with Cis (15 µM) for 24 h. Values are representative of three independent experiments. Each dot represents data from a single experiment. Error bars indicate SD *P < 0.05, **P < 0.01, ***P < 0.001, unpaired t-test

M8 stimulation of WT CaSki cells for 24 h reduced cell viability by approximately 40% (Fig. 3a), in good agreement with our previous results (Fig. 2a). On the other hand, 24-h treatment of WT CaSki cells with 15 µM Cis—estimated to be a sublethal dose by MTT assay (data not shown)—decreased cell viability by only 20% (Fig. 3a). However, when we treated cells with Cis + M8, we recorded a 60% loss of cell viability (Fig. 3a), indicating that activation of RIG-I potentiates Cis-mediated cell killing. This enhanced growth inhibition of the double vs single treatment was still observed at 48 h (60% reduction with Cis or M8 alone vs 90% with a combination of both), while at 72 h this effect was not statistically significant, probably due to the increased cytotoxicity of Cis over time. No statistically significant differences were observed between Cis alone and Cis + M8 in RIG-I KO CaSki at any time point, supporting a functional role of RIG-I in sensitizing cancer cells to chemotherapy. Furthermore, Cis + M8 treatment enhanced the percentage of apoptotic cells (41.5%) when compared to each treatment alone (11% and 12% in M8- or Cis-treated cells, respectively) (Fig. 3b). The percentage of Cis + M8-tretated RIG-I KO CaSki cells undergoing apoptosis was significantly lower than that of WT CaSki similarly treated (41.5% vs 12%), and no significant changes were observed between cells treated with Cis alone and those exposed to Cis + M8. Lastly, when we measured the release of IFNs and IL-6—with the latter being a prototype of inflammatory cytokines (18)—in these culture supernatants, we found that IL-6 release was significantly enhanced by the combination treatment when compared to each single compound: from 2 to 4 ng/mL in Cis- or M8-transfected cells vs almost 25 ng/mL in Cis + M8-transfected cells (Fig. 3c, left panel). In contrast, there were no significant differences in IFN release between cells treated with M8 + Cis and those with M8 alone (Fig. 3c, middle and right panels). As expected, in RIG-I KO CaSki cells, the release of all the three cytokines remained very similar to mock-transfected cells, confirming that RIG-I was required for their transcriptional activation (Fig. 3c).

Altogether, our findings show that M8 transfection in CaSki cells significantly enhances Cis-induced cell death and IL-6 release in a RIG-I dependent manner.

### M8 enhances the anticancer activity of cisplatin in a syngeneic HPV16-driven mouse cancer model

**Fig. 4:**
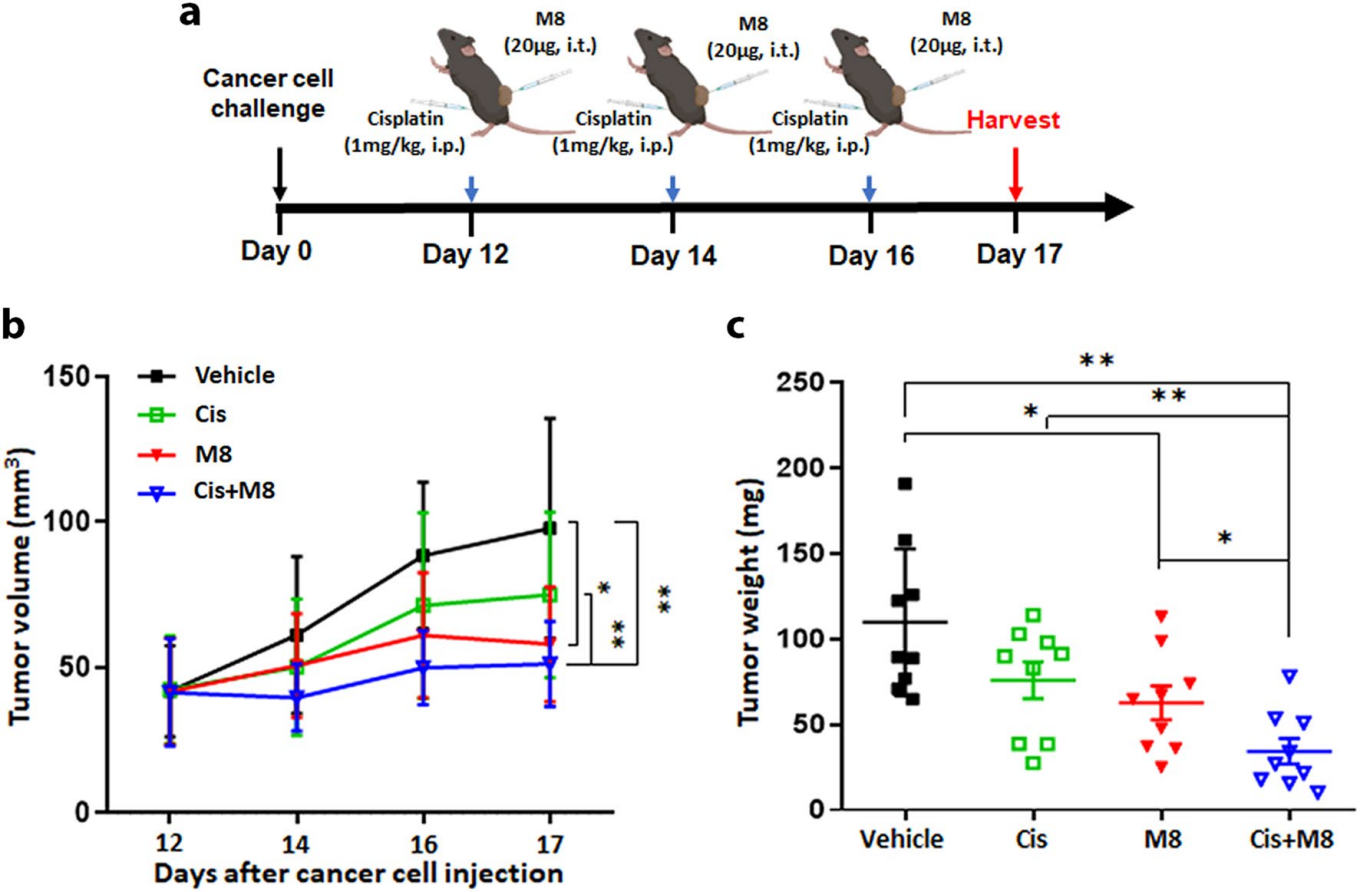
The RIG-I agonist M8 enhances cisplatin anticancer activity in an in vivo syngeneic mouse model of HPV16^+^ cancer **a** Schematic illustration of the tumor model in which C3.43 cells were subcutaneously inoculated into the back of C57BL/6 J mice. M8 (20 µg) and JetPEI (vehicle) were administered intratumorally, and Cis (1 mg/kg) intraperitoneally from day 12 after cancer cell injection, with one dose every two days up to day 16. Twenty-four h after the last treatment, the tumors were harvested. **b** Tumor volumes from mice (*n* = 9 for vehicle, *n* = 9 for M8, *n* = 9 for Cis, and *n* = 9 for M8 + Cis), treated as indicated in panel A, were measured every two days and recorded as mean volume of the tumors for each treatment.** c** Tumor weights from mice treated as indicated in panel A were determined at the endpoint of the experiment. Each dot represents data from a single mouse. Error bars indicate SD *P < 0.05, **P < 0.01, unpaired t-test, ***P < 0.001, unpaired t-test

We next asked whether the potentiation of Cis cytotoxicity by M8 in human cell lines would be reproducible in vivo. For this purpose, we used a syngeneic mouse tumor model obtained by dorsal subcutaneous (s.c.) injection of C3.43 cells, which harbor an integrated HPV16 genome, in C57BL/6 J mice [25]. After 12 days from the engraftment, when the tumors were approximately 50 mm^3^ in size, we started treating the animals (*n* = 9 mice for each treatment regimen) every 48 h, for a total of three administrations: 20 µg of M8 intratumorally (i.t.), 1 mg/kg of cisplatin intraperitoneally (i.p.), or a combination of both (Fig. 4a). We used JetPEI as vehicle to deliver M8 i.t. to focus immune activation to the tumor and its local environment. Both vehicle and i.t. administration have already been successfully used in other in vivo tumor studies [6, 8, 9]. The animals were killed after 24 h from the last treatment (day 17 from the inoculum), and tumors were harvested and weighted. As shown in Fig. 4b and c, when M8 and Cis were combined, we found a strong reduction in both tumor volume and tumor weight in comparison with the monotherapy groups or control mice. When we compared the impact of either M8 or Cis monotherapy on tumor burden, we found that only M8-mediated tumor inhibition was statistically significant when compared to vehicle-transfected mice (Fig. 4b, c).

Altogether, our findings in the in vivo model recapitulate those obtained in the in vitro model and further confirm that M8 strongly enhances the anticancer activity of Cis.

### M8 increases the recruitment of innate immunity cytotoxic effectors in a syngeneic HPV16-driven mouse cancer model

**Fig. 5:**
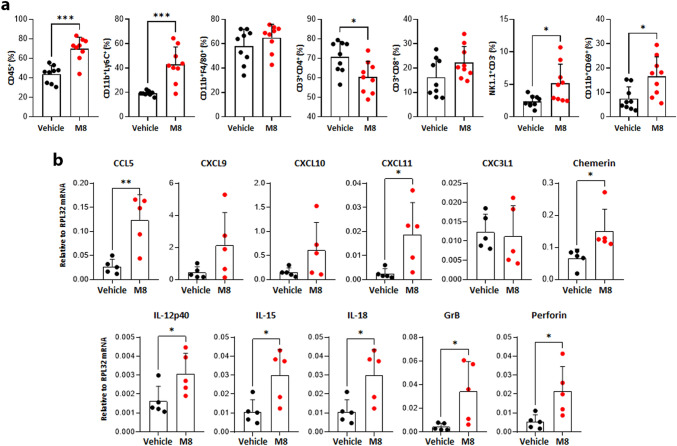
M8-treated tumors display increased numbers of activated NK cells **a** M8- or vehicle-treated tumors as described in Fig. 4a were harvested 24 h after the last treatment and dissociated. Subsequently, their immune cell infiltration was assessed by flow cytometry. Antibodies against the indicated antigens were used to distinguish the following cell populations: hematopoietic cells (CD45^+^), NK cells (NK1.1^+^/CD3^−^), monocytes (CD11b^+^Ly6C^+^) macrophages (CD11b^+^ F4/80^+^), T cells (CD3^+^CD4^+^ and CD3^+^C8^+^) and activated NK cells among total NK cells (CD11b^+^/CD69^+^). Each dot represents data from a single mouse. **b** Transcript levels of the indicated genes were assessed by qPCR using the total RNA extracted from the tumors treated as described in panel A. Data were normalized to the housekeeping RPL32 gene. Each dot represents data from a single mouse. Error bars indicate SD. *P < 0.05, **P < 0.01, ***P < 0.001, unpaired t-test

We next investigated whether the intratumoral administration of M8 may regulate tumor growth by impinging on the overall immune landscape within the tumor microenvironment. To this end, tumors harvested 24 h after the last M8 injection (day 17), were dissociated and the immune infiltrate analyzed by flow cytometry. As shown in Fig. 5a, compared to vehicle-treated tumors, M8 treatment induced a significant increase in the number of tumor-infiltrating CD45^+^ leukocytes (44% vs 70%), including CD11b^+^Ly6C^+^ monocytes (19% vs 43%), and NK1.1^+^CD3^−^ cells (2.4% vs 5.1%), whereas the proportion of CD4^+^CD3^+^ T cells was significantly decreased (71% vs 60%), and that of CD11b^+^F4/80^+^ macrophages slightly changed (59% vs 64%). Within the NK cell infiltrate, a higher percentage of NK cells expressing markers of both activation (CD69) and maturation (CD11b) was detected upon M8 treatment when compared to vehicle-treated tumors (7.5% vs 15.4%). The number of CD8^+^ CD3^+^ T cells was also slightly increased, albeit it did not reach statistical significance (16% vs 22%).

Having found enhanced NK cell recruitment upon M8 treatment, we decided to assess the intratumoral mRNA expression levels of the major NK cell-attracting chemokines (Fig. 5b). We observed a significant increase in CCL5 (4.5-fold), CXCL11 (9.5-fold), and chemerin (2.3-fold) mRNA expression levels. On the other hand, despite an upward tendency, changes in CXCL9 and CXCL10 mRNA expression levels never reached statistical significance. Finally, no change was observed for CXC3L1. In agreement with their role in NK cell activation, proliferation, and survival, the mRNA expression levels of the IL-12p40, IL-15, and IL-18 were also significantly increased in M8- vs vehicle-treated tumors (Fig. 5b) [34–36]. Finally, M8-treated tumors displayed a significant increase in granzyme B (GrB) and perforin mRNA expression levels, two cytotoxic cell effector genes. Thus, M8 treatment favors the creation of a tumor microenvironment enriched in NK cells, possibly depending on the increased expression of chemokines and cytokines that favor NK cell recruitment and activation.

### Conditioned medium from M8-transfected CaSki cells boosts NK cell proliferation, activation, and migration in vitro

Based on the in vivo data pointing to NK cell activation upon M8 i.t. injection, and assuming that the biological impact of this compound would be mostly due to a direct action on cancer cells and ensuing paracrine loop, we decided to analyze the activity of the conditioned medium from M8-transfected CaSki cells on NK cell activation in terms of proliferation, killing, and migration.

**Fig. 6:**
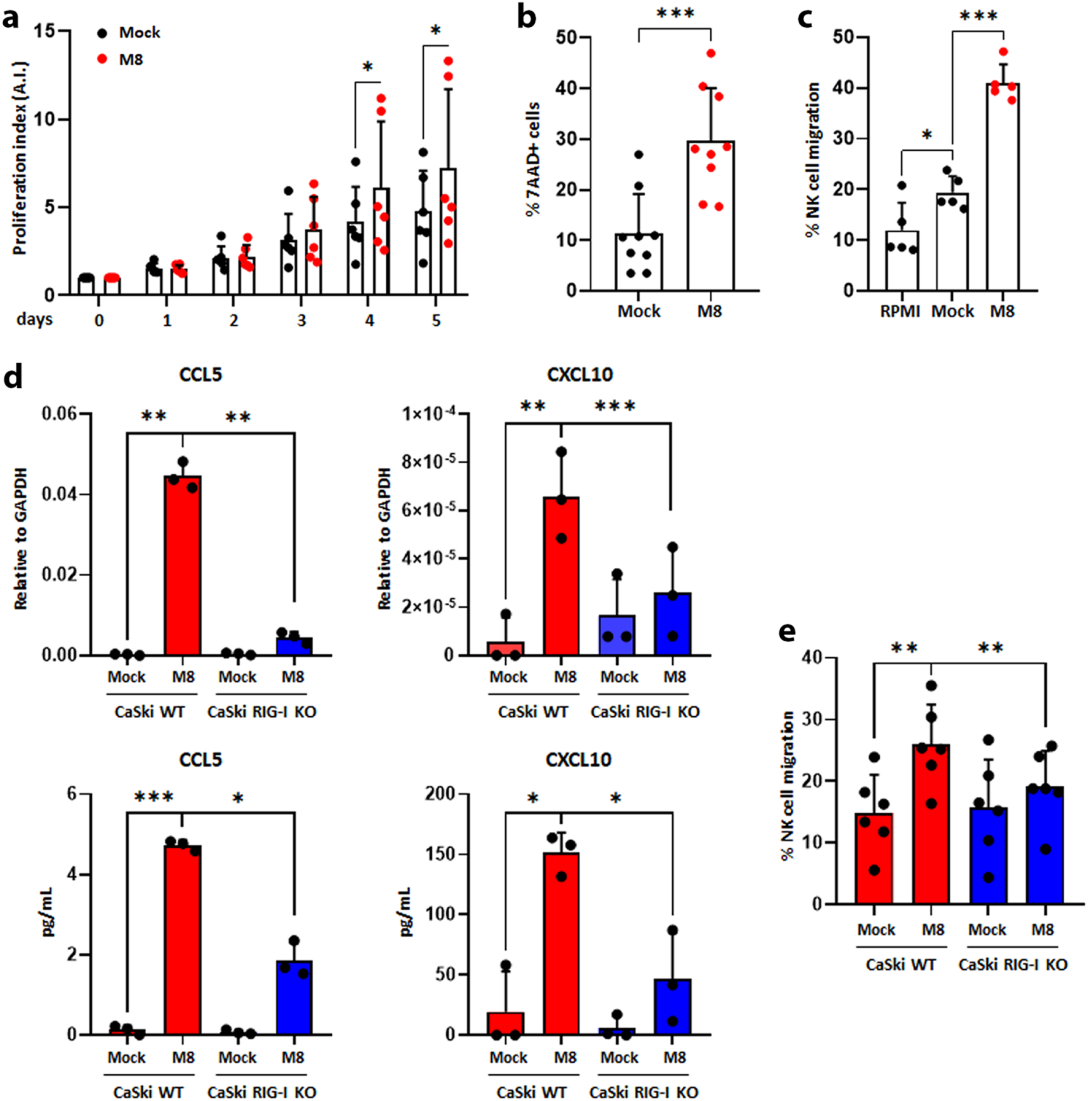
M8-transfected CaSki cells boost NK cells functions in a paracrine manner** a** Conditioned medium from M8- or mock-transfected CaSki cells (10 ng/mL of M8 for 24 h) was added to freshly purified NK cells from healthy donors. Proliferation was measured by Live-Cell Analysis and analyzed by using the IncuCyte® Zoom software. The proliferation index was calculated by setting to 1 NK cells at day 0. Pooled data are from five independent experiments with NK cells obtained from six different donors, and they are expressed as average of fold increase ± SD.** b** NK cells were incubated O/N with conditioned media as in panel A and then used as effectors in 7-AAD cytotoxicity assays against target CaSki cells. Data are from seven experiments with NK cells obtained from nine different donors at a 20:1 ratio. Values represent the percentage of 7-AAD positive cells from which the percentage obtained from target cells alone was subtracted. **c** Supernatants from the cells described in panel A were used to stimulate NK cells for 4 h, and then they were assessed in migration assay. The percentage of migrated cells was calculated as the number of migrated NK cells/number of input NK cells × 100. Data show mean ± SD from two independent experiments with NK cells obtained from five different healthy donors. All statistical analysis was performed with Student’s t-test. **d** Transcript levels of the indicated genes were assessed by qPCR using total RNA extracted from WT or RIG-I KO CaSki cells transfected or not with M8 (10 ng/mL). Data were normalized to the housekeeping GAPDH gene (upper panels). The supernatants were analyzed by ELISA for the presence of the indicated chemokines (lower panel. Each dot represents data from a single experiment, error bars indicate SD. **e** Freshly isolated NK cells were incubated with the supernatants from the cultures of panel D and then analyzed by migration assay as described in panel C. Data show mean ± SD from two independent experiments with NK cells obtained from six different donors. Statistical analysis was performed with Student’s t-test (*P < 0.05, **P < 0.01, ***P < 0.001; paired t-test)

Freshly isolated human NK cells were stimulated with conditioned supernatants from CaSki cells treated O/N in the presence or absence of 10 ng/mL of M8, and their proliferation rate monitored overtime by IncuCyte® Live-Cell Analysis. As shown in Fig. 6a, a statistically significant increase in cell proliferation was observed at days 4 and 5 in NK cell cultures grown in the conditioned medium from M8-transfected vs mock-transfected CaSki cells.

Next, we sought to determine the effect of these conditioned media on NK cell killing. To this end, freshly isolated NK cells were stimulated overnight with M8- or mock-transfected supernatants and then used as effector cells in 7-AAD cytotoxicity assays against CaSki target cells. NK cells stimulated with conditioned medium from M8-transfected CaSki cells displayed a threefold higher level of cytotoxicity when compared to that of NK cells challenged with mock-transfected supernatants **(**Fig. 6b). Lastly, the impact of the same conditioned media on NK cell migration was also analyzed. As shown in Fig. 6c, NK cells challenged with the supernatants from M8-transfected CaSki cells displayed an increased ability to migrate, ranging from 20% of migrated cells in response to mock-transfected medium to 40% when challenged with the conditioned media from M8-transfected CaSki cells.

As the findings from the in vivo model showed that M8 i.t. injection led to a significant enrichment in NK cells along with enhanced expression of key chemokines, very likely driving their migration [35, 37], we next assessed both mRNA expression and protein secretion levels of CCL5, CXCL9, CXCL10, and CXCL11 in M8- vs mock-transfected CaSki cells or in their RIG-I KO counterparts similarly treated. Interestingly, we found that CCL5 and CXCL10 (Fig. 6d), but not CXCL9 or CXCL11 (data not shown) were significantly augmented upon M8 transfection at both the mRNA and protein level (Fig. 6d), whereas their expression levels significantly dropped in RIG-I KO CaSki cells. As a further proof that the chemokines released by RIG-I stimulation were involved in enhanced NK cell migration, the conditioned media from the aforementioned cultures were used as chemo-attractants of freshly isolated NK cells (Fig. 6e). As expected, the number of migrating NK cells challenged with the media from M8-transfected CaSki cells significantly increased when compared to mock-transfected cells. Notably, the number of migrating NK cells was strongly reduced following stimulation with the media from M8-transfected RIG-I KO CaSki cells (Fig. 6e).

Altogether, our findings indicate that RIG-I stimulation by the 5’ppp-RNA M8 promotes intrinsic anticancer activity and stimulates NK cell migration in a paracrine fashion.

## Discussion

Current therapies for HPV-related cancer of the genital tract or head and neck region are often associated with significant treatment-related morbidity and/or toxicity. These treatments typically involve surgical excision, primary radiotherapy or chemoradiotherapy, all with devastating effects on the targeted anatomical sites [14–17, 38]. The prevalence of these tumors in younger individuals, especially OPSCC, emphasizes the need to devise new therapies that can ensure a high post-treatment quality of life (QoL). Owing to the vulnerability of HPV-associated cancers to antitumor immunity, it is highly likely that immunotherapies will become the centerpiece of future combination therapies aimed to reduce morbidities and mortality associated with the current standard of care for these tumors.

Although RIG-I signaling has been involved in anticancer immunity in several tumors, such as pancreatic cancer, hepatocellular carcinoma, leukemia, breast cancer, and melanoma, its role in HPV-associated cancers is still unknown [6–10]. Here, we demonstrate the feasibility of a novel combinatorial therapeutic approach against HPV-associated cancers based on cancer cell-intrinsic cell death triggered by drug-targeted activation of the RIG-I pathway, which in turn potentiates existing anticancer therapies. Specifically, we show that activation of RIG-I signaling through transfection of the RIG-I agonist M8 promotes growth arrest and apoptosis of two HPV^+^ human cervical carcinoma-derived cell lines. This inhibitory activity is significantly, but not entirely, reversed in RIG-I KO cells, suggesting that M8 may also act through different pathways. In light of these results, it will be extremely important to extend our findings to other anatomical sites with stratified squamous epithelia that can also develop cancer due to HPV infection (e.g., the head and neck region). This would require using cell lines derived from human carcinomas of these sites.

Another key finding from this study is the demonstration that M8 treatment enhances the anticancer activity of the chemotherapeutic agent Cis, which is routinely used to treat HPV-associated cancer [31, 38]. These in vitro results were readily reproduced in a syngeneic mouse model of HPV16-induced cancer where i.t. injection of M8 potentiated the anticancer activity of Cis [25]. These findings together with the observation that the *DDX58/RIG-I* gene seems to be rarely mutated in HPV-associated cancer suggests that RIG‐I activation may constitute an effective treatment strategy against this type of cancer.

Leveraging RIG-I signaling to induce cell death of HPV^+^ cancer cells is an approach further supported by the well-established notion that this pathway is functional in these cells [19, 21, 22]. Indeed, when RIG-I is stimulated with powerful agonists (e.g., M8), it can boost the transcription and secretion of several IFNs, especially type III IFNs. By contrast, the DNA sensing pathway that involves the cGAS-STING signaling pathways has been shown to be heavily impaired in HPV^+^ cancer cells through multiple inhibitory mechanisms involving the viral oncoproteins E6 and E7, and thus hardly reactivatable, in both in vivo and in vitro models of HPV-induced cancer [20–23]. Therefore, specific targeting of the RIG-I pathway appears to be an attractive and feasible option to trigger the innate immune response in HPV^+^ cells.

The ability of the RIG-I agonist M8 to push cells toward a pro-inflammatory phenotype through the production of type I IFNs and lymphocyte-recruiting chemokines makes this compound a suitable candidate to enhance cell death in HPV^+^ cancer cells and improve the effectiveness of existing anticancer therapies against HPV-associated cancer. In keeping with previous studies showing that treatment with other RIG-I agonists induced cancer cell death mediated by immune cells, including DCs, NK cells, and CD8^+^ T cells [39–42], we show that i.t. injection of M8 increases the proportion of infiltrating CD45^+^ leukocytes. Among these cells, we found a significant increase in the number of activated NK cells, as judged by the increased percentage of NK cells expressing the activation markers CD11b and CD69, along with enhanced mRNA expression levels of the cytotoxic cell effector genes granzyme B and perforin. Consistent with the observed changes in the tumor microenvironment, a series of chemokines and cytokines known to be involved in NK cell recruitment and activation [34, 35] were found to be significantly transcriptionally upregulated upon M8 stimulation. However, we recognize a limitation of this study in not performing single cell transcriptome analysis that would have aided in better dissecting the activation status of the different immune infiltrate cells.

The creation of an inflamed microenvironment in the tumors following M8 exposure was also demonstrated in vitro using the HPV^+^ CaSki cervical carcinoma-derived cell line. In particular, exposing freshly isolated NK cells to conditioned media from M8-transfected CaSki cells increased NK cell proliferation, killing of CaSki target cells, and migration. Notably, M8-transfected CaSki cells expressed enhanced levels of the NK-attracting CCL5 and CXCL10 chemokines [37]. It is also worth mentioning that the transcriptional activation and secretion of these chemokines upon M8 transfection was RIG-I dependent as their expression significantly dropped in RIG-I depleted CaSki cells. Accordingly, the conditioned media from M8-transfected RIG-I KO CaSki cells failed to boost NK cell migration when compared to the media from M8-transfected parental CaSki cells.

In summary, our findings shed light on a previously unrecognized role of the RIG-I agonist M8 as a potential therapeutic option to kill HPV^+^ cancer cells and provide the proof-of-concept that RIG-I triggering by M8 can be used in combination with standard chemotherapies to enhance their anticancer efficacy.

In addition to intrinsic-cancer cell death, M8 transfection of HPV^+^ cancer cells was also found to boost NK cell activation and killing in a paracrine manner. Indeed, a panel of chemokines, including CCL5 and CXCL10 were found to be upregulated in both M8 treated CaSki cells or tumors.

NK cells have been shown to play a crucial role in host defense against viral infections and malignancies, including HPV-driven cancer. Patients with inherited NK functional defects display enhanced susceptibility to this type of cancer, and a higher proportion of activated NK cells in HPV^+^ tumors contribute to a favorable outcome [43–47]. Thus, the possibility to boost NK cell recruitment and activation in HPV^+^ tumors via RIG-I triggering with M8 may allow the development of alternative antiviral/anticancer therapies. Furthermore, given the additive effect of M8 and Cis observed in both in vivo and in vitro experiments, it is possible that co-administration of these agents may allow the design of de-escalating anticancer regimens, which would spare patients with treatment-related chemo- and radiotoxicity [32, 33, 48]. Finally, it is important to point out that intratumoral delivery of M8 in humans may be a feasible procedure given that HPV-associated lesions are usually localized in easily accessible sites in the anogenital region and the upper aerodigestive tract, with the latter taking advantage of newly developed minimally invasive transoral surgical techniques.

In conclusion, our findings emphasize the potential for treatment de-intensification strategies based on M8-mediated activation of RIG-I, which would ultimately improve the QoL of patients undergoing therapies against HPV-associated cancers by minimizing long-term morbidity.

## Data Availability

Data are available upon request.
